# Selections and Abstracts

**Published:** 1898-06

**Authors:** 


### SELECTIONS AND ABSTRACTS.

### THE MODIFICATION OF COW'S MILK.

The first object to be attained in the modification of cow's milk is to secure the proper quantities of the albuminoids, fat, and sugar, so that when combined it shall be similar to human milk. The ingredients used are milk, cream, sugar of milk, water, and lime water. A misapprehension exists as to what cream really is. It is simply a milk rich in fats, containing about 16 per cent. of fat, 4 of albuminoids, 4 of sugar. This is gravity, or skimmed, cream taken from milk that has stood at least twelve hours. It is always better to procure the milk and skim the cream rather than to buy the cream, because cream that is 16 per cent. fat is very much richer than that procured in the market. Sugar of milk can now be purchased at a very reasonable rate, in quantities of one pound or more. One pound of sugar of milk is sufficient for sixteen pints of prepared food. Allowing two pints for each twenty-four hours, which is the average for a baby of six months, it will be seen that this does not make an expensive food. Indeed, the expense is less than where patent foods are used. It should be understood that the milk used in preparing the food is fresh unskimmed milk, and during the summer season the food should be prepared twice each day, so that each time fresh milk is used and the cream from milk that has stood for twelve hours. In mixing this food certain articles are necessary: A graduated glass of a capacity of six or eight ounces, a measure for the milk sugar. This measure should hold about one ounce, by weight, of milk sugar. It may be of glass, china, or tin, and by getting a sample package of one ounce of milk sugar and placing it in the receptacle and marking it, it may be used for each subsequent time. A glass jar of the capacity of one pint, in which all the ingredients are mixed. The following formula will be found suitable in the majority of case:

| | |
|---|---|
| Milk | 2 ℥ |
| Cream | 3 ℥ |
| Milk sugar | 1 ℥ |
| Water | 10 ℥ |

In preparing this it is better to measure the 10 ounces of water into the glass jar, add the milk sugar and stir until the solution is made, then add the cream and milk and stir gently. When all the ingredients are placed in the jar and it is agitated until the sugar is in a state of solution, the cream is very likely to be more or less churned and the fat globules separated. Having properly combined these elements, the first indication—that is, a food similar to human milk—has been met, with the exception of the lime water, which should not be added until after the food is sterilized, the reason for this being that some chemical change probably takes place in the process of heating. This food may now be sterilized either in bulk or in individual nursing-bottles, as the case may be. The latter method is the better in that it avoids the contamination of the food in emptying it from the large vessel into the nursing-bottle, but with care it may be sterilized in pint quantities with comparative safety. The food is sterilized, not to make it more digestible, but to destroy pathogenic bacteria. Were it possible to get a pure milk supply and keep it so until ready for use, it would probably be better if it were never sterilized at all; but as that is out of the question, and as the danger of infection is so great, we choose the lesser of two evils and sterilize. Experience has taught that the prolonged heating of the milk at a high temperature, while destroying pathogenic organisms, is objectionable in that it renders the milk less digestible and more constipating. The more recent practice of pasteurizing is less objectionable, and, at the same time, may be safely depended upon to destroy most of the pathogenic germs found in milk.

By *pasteurization* we mean the heating of it to about 170° and maintaining that heat for twenty minutes. In private practice where people cannot be supplied with expensive apparatus, thermometers, etc., we can only approximate the degree of heat. Where the Arnold sterilizer can be procured it is a very simple matter, as the bottles are contained in a steam chamber which is probably always below the boiling point. Where this cannot be procured, it is better to direct the mother to place the bottle or bottles in a tin bucket having a false bottom, beneath which a small quantity of water is placed and allowed to boil, so that the milk contained is in an atmosphere of steam. We probably rely too much upon pasteurization of milk and too little upon a pure milk supply. Evidence is accumulating to demonstrate that impure milk when pasteurized is not safe; that while the germs may be destroyed, the activity of the ptomaines already generated are not destroyed by heating, and that certain cases of acute sickness are probably due to ptomaines in the milk rather than to ptomaines developed in the body. This distinction should be borne in mind in our investigation of the cause of acute sickness in bottle babies.

After the milk is pasteurized lime water can be added, either to the food in bulk or to the individual nursing-bottles. One ounce of lime water to the pint is sufficient to render the milk alkaline in reaction.

Having procured a milk containing the proper chemical constituents and having it pasteurized, the next problem is that it may be conveyed to the stomach of the child in this sterilized condition. We should never forget that the nursing-bottle and nipples may themselves be sources of infection. Too much care cannot be exercised in the washing of the bottles and nipples. Simple washing of the bottles is not sufficient. They should, especially in the summer, after being thoroughly washed, be placed in a vessel of water and thus boiled for twenty minutes or more, in order that the botles themselves may be completely sterilized. The nipples should be carefully cleansed inside and out, and when not in use kept in an alkaline solution of either borax or soda. The nipples with rubber tubes are an abomination and should be discarded. In young infants it is important that the food should be of the proper temperature, neither too hot nor too cold. Probably the most convenient way is to place the nursing-bottle containing the proper quantity of milk for one feeding in a pan of water as hot as can be borne by the hand, and permit it to remain there until the bottle is heated through.

The formula given will not suit all cases, but the advantages are that the percentages of the various elements can be easily changed,

*e. g.*, where a child vomits this milk in hard curds we may reduce the amount of albuminoids by entirely withdrawing the milk and by slightly increasing the cream; *e. g.*, 4 ounces of cream, 12 ounces of water, and 1 ounce of milk sugar. This makes a nourishing food, and yet more easy of digestion. The greatest difficulty will be found in giving to children that have been badly fed for several months, especially where they have had a food deficient in fats and proteids and where, as a result, they have taken large quantities. It will usually be found that a child upon condensed milk will take five or six ounces when it should be taking two or three ounces, and when placed upon this mixture it will still take excessive quantities and an indigestion result, not from the fault of the food, but from the abnormal condition of the child. In cases of this kind it is better to still further dilute the food; *e. g.*, take four ounces of this food and add two ounces of sterilized water, and then attempt gradually to reduce the quantity of food and withdraw the surplus of water.

*Regularity of Feeding.*—Much depends upon this, and with a little care a child can be trained to nurse from the bottle at regular intervals. During the first three months of life an infant should nurse every two hours during the day and once in the middle of the night, or about ten nursings in twenty-four hours, and the quantity of milk at each nursing should not exceed three ounces. From three to six months the child should nurse about every three hours during the day, or seven nursings during the twenty-four hours, and should take four or five ounces at each nursing. From six to ten months the child should nurse about every four hours and take six to eight ounces at each feeding. The tests of a food are, first, whether or not it agrees with the patient, and, second, whether or not the patient gains in weight. A steady gain in weight is the most important index as to whether the food is agreeing with the child and whether the child is thriving properly. During the first six months of life infants should gain on an average of half a pound a week. Bottle-fed babies are more prone to attacks of indigestion than nursed babies, and these attacks are very frequently due to some fault with the feeding, either too large a quantity of food or too rich a food, or food given too hot or too cold. Rather than prescribe medicines it would be better for the patient if the physician would carefully look into the methods of feeding for the cause, and it will usually be found that by changing the proportion of the elements this difficulty can be overcome. Constipation is one of the most annoying conditions and the most difficult to correct. Frequently, by increasing the cream we will aid in overcoming the constipation. Another simple method is to give water freely between nursings. Where the various modifications of cow's milk fail, as is sometimes the case, owing to the inherent differences in the albuminoids of human and cow's milk, we may then predigest the cow's milk. By this process the albuminoids are converted into soluble peptones.

In the following I quote from Professor Albert R. Leeds: "Take of milk half pint, water half pint, cream four tablespoonfuls, peptogenic milk powder one measure. This mixture is heated on a hot range or gas stove, with constant stirring, the heating being so conducted that at the end of ten minutes it is brought to the boiling point. This is put in a clean bottle, corked, and placed on ice or in cold water until ready for use." Peptogenic milk powder contains pancreatic extract, lactose, and alkalies, and the "measure" is found with every package.—DR. MCCLANAHAN in *Western Med. Review.*

---

### SOME POINTS IN THE EXAMINATION FOR LIFE INSURANCE.

At first the physique and general appearance should be noted. Are the eyes bright; is the complexion a healthy one; is there any puffiness under the eyes or on back of hands; or swelling of feet or ankles; is there any lameness in walking.

In making a physical examination of the lungs and heart the outer shirt should be removed, for if it contains starch, on a deep inspiration it produces a crackling sound simulating crepitant or subcrepitant râles. Inspection should be made of the chest with reference to fulness of intercostal spaces, and undue prominence of chest or contraction, or depression under the clavicle—any of these may be significant of an old pleurisy, emphysema, phthisis and pericarditis. Auscultation, as a rule, should be made with the ear applied to the chest walls with a thin covering, as a towel. The examination should cover supra- and infra-clavicular region, supra- and infra-axillary region, and posterior over inter- and intra-scapular region. Inspection is of importance in examination of the heart; from it we determine the apex beat, force of beat, or change of beat. Palpitation is of great importance; by it we determine the force of the cardiac pulsation, the frequency or slowness of the heart's action and the irregularity of its movements. In auscultation, we place the ear over different valves of the heart and listen to the heart sounds while applicant is holding his breath, then direct him to breathe naturally, and finally tell him to take a few forced inspirations. By this method, if there be a murmur, it can be easily detected; in cases of doubt, the stethoscope may be used. The examination of the pulse is of great importance, and should be taken two or three times during an examination, and at each time for a minute. The following conditions should be noted:

Frequency of beats in a given time, regularity, intermittence, strength and force of the beats, is it compressible, or is it small and thready? With a pulse of high arterial tension the vessels are contracted and the blood escapes with difficulty from the arteries into the veins; the artery is cord-like and can be traced in its course up the forearm. This condition is often found in Bright's disease, in gout, affections of the nervous system, and in degeneration of vessels.

In arterial degeneration the vessel loses its elasticity, its lumen is diminished, and it becomes hard and rigid, or "pipe-stem artery." This is a very significant condition, being evidence of senile decay of the arteries. Many persons are constitutionally much older than their years will warrant—they are, in fact, prematurely old; while, on the other hand, many old people show few signs of old age.

It might be well to mention cases of alleged syphilis, for I have seen injustice done to the applicant in a great many cases. As a rule, applicants know nothing of the constitutional symptoms of syphilis, and if they have had a chancre and have fallen into the hands of a charlatan, he, of course, has syphilis, and goes on record as such, when, in fact, in many instances, he has had a chancroid and not the infecting chancre. In these cases examination of the cervical glands should be made, and an inquiry into the constitutional symptoms—*e. g.*: eruption, sore throat and fever. In many of these cases inquiry will reveal that applicant had none of the symptoms mentioned—and the physician burned the chancre and it got well.

Lastly the examination of the urine should receive our attention. It is embarrassing to say that, as a rule, examiners do not seem to realize the importance of making a careful analysis of the urine. Examiners should become familiar with a few of the most trustworthy tests and be capable of making a microscopical examination. In many instances the urine is the index that points to lesions that are just beginning. The centrifuge is as necessary for daily use as the standard solutions for chemical test; it can be used for quantitative estimation, and in sedimentation for microscopical examination. My experience has taught me that the specific gravity cannot be relied upon, and I have known of many who rely upon the specific gravity and make no further analysis of the urine, taking it for granted that 1,020 meant normal urine when, in fact, albumin and sugar may be present with a specific gravity at 1,020. Normal urine may range in specific gravity from 1,000 to 1,030; the food eaten, exercise and the amount of water imbibed—all will change the specific gravity of the urine without altering in the least the healthy condition of the kidneys. The examiner should, at least, become familiar with two tests for albumin and two for sugar. The tests that I use, and the ones I consider the simplest and the most delicate in reaction, are:

For albumin, a ten per cent. solution of potassium ferrocyanide, and Heller's nitric acid test.

For sugar I prefer one devised by Professor Wesener of Chicago, consisting of cuprum sulphate, two drachms; stick potash, six drachms; glycerine, one ounce; pure water, enough to make eight ounces. Also one devised by Professor Haines of Chicago, prepared as follows: Cuprum sulphate, thirty grains; pure water, one-half ounce; glycerine, one-half ounce; liquor potassa, five ounces; either one of these solutions will keep and is very delicate in reaction.

In examining the urine one should know, without any doubt, that the urine was voided by the applicant a few hours after breakfast, and the urine should be allowed to cool before being tested. A careful observation should be made of its appearance and physical character—if the color be very light, it suggests a diminished specific gravity; if the color be of greenish tint, it suggests the presence of sugar; if of a reddish tint, urates of blood is inferred. If the urine is cloudy, add a few drops of acetic acid, and if it becomes clear, the earthy phosphates were the cause of the opacity. If the opacity of the urine fails to yield to the action of the acid, warm the upper layers of the urine by holding the test-tube over a spirit flame, and if it now clears up, the opacity was due to urates. If, however, the urine still remains cloudy, it is due to the presence of pus, bacteria, or cellular elements, and requires a microscopical examination for diagnostic purposes. If the reaction is found to be sharply acid, as indicated by turning blue litmus red, the possibility of sugar is suggested. If the red litmus turns blue, the urine is alkaline; it is of importance to know the cause of the alkalinity to determine if there be a bladder trouble or alkalinity of the blood. These conditions may be solved by slowly drying the litmus paper and if the blue color disappears, and if it returns to its original color red, ammonia is present, or volatile alkali, and suggests chronic inflammation of the bladder or urinary tract. If, on the other hand, the blue color remains after drying, the urine is alkaline from fixed alkali and may not mean other than fasting or the absence of a meat diet. This condition I have seen so many times during warm weather when a minimum amount of meat was used in the diet, but when beef was ordered the urine would become naturally acid.

If albumin appears in large quantity, any of the ordinary tests for albumin will make it apparent; but if a small quantity is present, the test that will make it apparent is the one to use. For this reason I prefer a ten per cent. solution of potassium ferrocyanide. Fill a test-tube half full of urine, then add ten or fifteen drops of acetic acid, then add twenty or thirty drops of the ferrocyanide solution—if albumin is present a milky color will appear and spread through all the urine; by shaking the test-tube a few times the white color will appear more quickly. This test will only detect serum albumin and is not a source of error as are other tests for albumin. Heller's nitric acid test, is familiar to all; suffice it to say that this test is liable to be a source of error as it will give the albuminous reaction with other substances than serum albumin.

In testing for sugar I prefer the test devised by Professor Wesener, the formula of which is given above. The use of this test is simple: in a test-tube heat over a spirit flame to boiling point equal parts of urine and the test solution, if sugar is present the urine is changed to a brick-dust color; this test is delicate and will keep well.

The test of Professor Haines is a reliable one also. Place one drachm of the test solution in a test-tube, raise to the boiling point, then add four or five drops of the urine, continue the boiling and keep adding, drop by drop, until a change takes place to a brick-dust color; continue the adding of the urine until ten drops are added, then cease. Sometimes the reaction will take place when only a few drops are added; or it may not change until the ten drops are added, and if not then, sugar is not present.

In all cases, where albumin is found, a test for urea should follow. By testing for urea we are then able to judge the real condition of the kidneys, while albumin may not mean anything if the urea is normal in amount. In testing for urea, fill Doremus's ureometer with a fifty per cent. strength of caustic potash, add one c. c. of bromide and mix, incline tube so that solution fills it perfectly, then add one c. c. of the urine to the solution. The urea will be decomposed to nitrogen, then read off per cent. from above downward. The normal amount of urea passed by a man weighing 150 pounds, with a moderate diet and exercise, is ten to twelve grains per ounce. If below seven grains per ounce there is reason to suspect organic disease of the kidneys.—DR. L. P. WALBRIDGE, in *Medical Examiner*.

---

### CLEAN SURGERY IN MINOR AND EMERGENCY CASES.

The *Virginia Medical Semi-Monthly* contains a paper by Dr. Howard, of Alexandria, on the importance of clean surgery in minor and emergency cases of the general practitioner.

I do not intend to enter into a discussion or consideration of the various antiseptics or of the rationale of their action, but, accepting authority, and having urged and pleaded for their application, to now suggest some practical hints, by attention to which clean surgery may be obtained in minor work, and having become a *habit* in this class of cases, will be assured in such major work as the general practitioner is called upon to perform.

First, then, concerning minor work.

Your hands, though spotless, soft and white, and but just washed and dried on a freshly laundered towel, may be surgically filthy. Adopt some one of the various accepted methods for cleansing them, as bichloride, permanganate and oxalic acid, etc., but don't dry them afterwards on a non-sterile towel, and don't cleanse the nails with a knife which may not be clean any more than you have scrubbed them with a brush unclean from its last use.

Remember that "rinsing" in bichloride is a farce—laughable, but that it is criminal.

Don't grudge the time necessary to prepare to do "clean" work, as in the preparation of your "tools," viz.: instruments, needles, sutures, solutions, applications, and dressings.

All instruments should be sterilized twice after each operation and before. Boiling in soda solution [ʒjss–Oij] is as good a method as you can use. Boil for five minutes. If you cannot boil an instrument, passing through any flame will sterilize it; fresh soot is not unclean surgically.

Having cleaned your instruments and hands, do not soil them by contact with an unclean site of operation; this should be as carefully cleansed as your hands, by scrubbing and the use of some accepted antiseptic. Remember, bichloride will not act in the presence of grease, and that alcohol or ether will remove that substance.

Hands, instruments and sight of operation being now clean, viz., sterile, do not soil them by contact with immaculate towels, bedclothing, or portions of your own or patient's body not prepared for operation; let not that which is clean touch anything that is unclean; use only sterilized water for irrigation in nonseptic cases. Rely more on amount of the irrigation fluid used in septic cases—that is, on its mechanical antiseptic action rather than its chemical properties, and aid its thorough application by force of stream and, where feasible, by "rubbing it in." Don't irrigate from a fountain syringe nozzle which has not been boiled since last used, and don't hang nozzle in mouth of bag when not in use; if, by mistake, you have done this in a septic case, don't use bag again until sterilized.

Don't use a pocket case which you cannot sterilize; the leather velvet-lined case is an "omnium gatherum microbium." Don't use an instrument having a wooden handle until you have removed the wood; the best handles are flat with square edges.

Don't be stingy with the number of sutures, nor of your patient's patience in placing them; consider that you are to be judged by cosmetic effect.

Don't use adhesive strips, or, if at all, not so as to touch wound; they are always of doubtful cleanliness. Don't use iodoform or other dusting powder that you have not sterilized by "cooking," and don't consider expense in its use.

Don't be stingy with your gauze and absorbent; of the former, plain as good as any, but don't rely on the reputation of the house that bottled or boxed it—"cook" it yourself.

Don't think that absorbent from pound package is made clean for swabs by *dipping* in bichloride. Sterilize them beforehand by boiling or cooking.

Don't consider any wound too small for the entrance of the death-bearing agencies, or that slight pain and inflammation do not matter; they are infallible witnesses of carelessness and disregard of duty.

Don't dress your wound under five days, unless throbbing pain, fever or redness be present, or unless you have used drainage, which do only when you cannot be sure of your wound being clean.

Don't be ashamed of being considered by any as finicky; *quiet* attention to detail need not involve fussiness. Consciousness of duty performed will well repay for the sneer that you are making a big show; for a small, neat scar, soon to disappear, and a painless result, will in time convince the layman that it is not so much what you have done as the way you have done it.

I have finished with Don'ts. I have but one Do. Do be clean.

Asepticism, clean surgery, to be effective, must be perfect, or it is useless. It is only to be attained by absolute and minute attention to detail. One streptococcus pyogenes is only 0 7 mm. in diameter, is autogenous, and will, if applied with suitable pabulum and conditions, beget in twenty-four hours a family which would fill the Atlantic.

So much for clean surgery in minor work. We come next to such cases of major surgery as he may have to undertake under circumstances and surroundings less favorable than those which he could command for his minor work.

Such attention to cleanliness as we have suggested under minor work may best be attained for emergency cases, so far as your instruments, applications, dressings, hands and site of operation are concerned, by your providing yourself with an emergency bag; and it will well repay you the slight outlay required, not only in a pecuniary sense, but in others which I have in the early part of this paper touched upon. And let me here urge most earnestly that it is as much your duty to possess such a bag and to have it always with you ready for use, as it is to have a pair of obstetrical forceps; I am speaking now more especially to the country doctor. There are many emergency satchels now on the market—some, perhaps all, beyond the means of some of our ill-paid number. But surely there is not one of us who could not afford a bag say six by ten inches, in which were two scalpels, one dozen hemostats, one or more pairs of strong scissors, with aseptic lock, three sizes of Murphy's buttons (in case of strangulated hernia requiring resection), a carefully selected two dozen needles in a wide-mouth bottle (two of these needles of a size suitable for Czerny's sutures), one box of sterilized iodoform, six feet of rubber tubing, a half pound of absorbent cotton, one bottle of drainage tubes, one bottle of bichloride tablets, three two-ounce packages of plain gauze (cooked), four ounces of ether or chloroform, two ounces of peroxide, one trephine and elevator, all carefully wrapped in a sterilized towel, and six small sterilized towels, wrapped in oiled paper or silk, laid on top.

### MODERN GUNSHOT WOUNDS.

In these times of war there is an added interest to the consideration of the injuries inflicted by the weapons of modern warfare, a subject that even in peace forms a branch of the department of surgery. At this time of writing there has been no engagement of land forces, and no casualties of consequence of any kind; but since the introduction of the most recent models of small arms there have been a sufficient number of engagements in various parts of the world to make it possible to predict with some accuracy the kind of wounds with which the army surgeon will have to deal while the troops are engaged in driving the Spaniards out of Cuba.

So rare are sword cuts and bayonet stabs in modern battles that wounds from these sources form an inconsiderable part of the casualties of the battle-field. Missiles from the artillery do a good deal of damage, but the character of this class of wounds has not changed. Almost all the injuries inflicted in battle come from rifle balls, and in this firearm there have been great changes within a few years, the caliber of the weapon in this and other countries having been greatly reduced, while the range has been enormously increased by giving the bullet a high initial velocity. Another recent feature which greatly modifies the character of gunshot wounds is the jacketing of the leaden bullet with a harder metal, such as nickel or German silver, preventing deformity of the missile even after contact with hard substances. The United States has adopted as the regulation arm the Krag-Jorgenson rifle, which has a caliber of 30 one-hundredths of an inch. As the supply on hand of this weapon amounts to but one hundred thousand, only the regulars will be armed with this rifle, the volunteers being provided with the Springfield rifle of 45 caliber. The Spanish troops are likewise furnished with two kinds of rifles, the Mauser of 7 m. m. caliber, equal to about 28 one-hundredths of an inch, and the Remington army rifle of 43 caliber. It will thus be seen that the armies about to oppose one another in Cuba will be on a practical equality in the matter of firearms, and that the wounds inflicted on the two sides will be much the same in character.

At very short ranges the small caliber ball with high velocity produces an effect described as "explosive," the injury to the tissues being so great and so widely spread that when these wounds were first observed in battle the enemy were accused of using explosive bullets. Explosive effects are noted particularly in the brain and liver, these organs being largely destroyed by the passage through them of a single bullet. In the soft parts of limbs the destruction of tissue is described as "frightful," while the long bones are fractured and comminuted to an astonishing extent, the splinters and fragments of bone being driven to great distances.

The limit of range at which explosive effects are observed is variously stated by different observers. In experiments made upon cadavers at the Frankford arsenal in Philadelphia, the range was given as 250 yards for the 45 caliber rifle and 350 yards for the 30 caliber.

At greater distance, up to 2,000 yards, covering the ranges at which the majority of wounds are inflicted on the battle-field, the small caliber ball of high velocity produces less severe wounds than did its larger and slower predecessors. This is particularly true of the jacketed bullet, that does not become flattened or "mushroomed" on striking a hard substance. The small, swift-moving bullet perforates the skin with a round, narrow orifice with clean edges. In fibrous tissue it makes a narrow slit; in muscle a fistulous track a little larger than itself; in bone a hole sometimes as cleanly cut as if punched out, sometimes with splintered edges, according to the velocity. An important difference between the old and the new wounds is where an artery is involved. The danger of hemorrhage upon the battle-field used to be lightly esteemed, but the bullet with high velocity cuts arteries as sharply as does the knife, and there is no doubt that the danger from primary hemorrhage will be greatly increased.

Beyond the limit of its explosive action, the bullet from the new rifle justifies the claim made for it that as compared with the old rifle ball it inflicts wounds less severe and more easily recovered from. Wounds of soft tissues, even of the lungs themselves, are found to be much diminished in severity. Indeed, the mildness of the injury it inflicts is urged as a serious objection to the new rifle. The object of the soldier is to disable the enemy as quickly as possible. In general, the shock of even a flesh wound may be depended upon to prevent a man from taking further part in a battle, but the swift-moving, small-caliber bullet, with a hardened case preventing its deformity, produces so little shock that the stopping power of the new rifle is comparatively small, and there is a great question as to its effectiveness in stopping a rush of fanatical and barbarous men like the Dervishes of the Soudan or the Abyssinians. The shock to horses is also so much less that it is doubtful if the new rifle can be depended upon to stop a cavalry charge as did the old weapon. Without reference to the part hit, the shock depends upon the loss of energy of the ball in its passage through the body. As it is evident that a bullet that passes through the body undeformed will lose much less energy than one that becomes "mushroomed" on the way, experiments have been made upon the use of bullets with soft tops that will spread out when they meet with an obstacle, and be so slowed as to increase the amount of shock produced.—*Northwestern Lancet.*

---

### CURETTAGE AND PACKING THE UTERUS.

J. Duncan Emmett (*Am. Gyn. and Obstet. Jour.*, May) takes exception to the general teaching that endometritis is an important or even a common factor in inflammatory diseases of the pelvis. In the popular medical mind to-day the use of the curette implies a belief in the doctrine of endometritis as the *fons et origo*—the foundation-stone, in fact—of the diseases of women. This is neither just nor true, in the opinion of the author. In the understanding of Dr. Polk, to whom is given the chief credit for the revival of curettage and drainage, at least in the sense of popularizing it, and in that of the vast majority of its present supporters, the chief significance of this operation lies in the claim that it is curative of endometritis. This is defined as an inflammatory disease, *per se,* of the lining membrane of the uterine canal. Its origin is by extension through the vagina, extension through the tubes, or by the introduction of septic matter by means of the lymphatics. Its clinical symptoms are uterine "leucorrhea" and enlargement, with tenderness in and around the uterus. If endometritis in its acute form be considered as a primary and independent disease, as its advocates claim it to be, we should, in the estimation of the author, expect some evidences of distinct inflammation in the endometrium itself; yet we never find this, but the whole uterus as well as the peri-uterine tissues show equal evidences of inflammation. In acute endometritis, therefore, it is pure assumption to claim that the metritis and perimetritis are secondary diseases in regard to it. In chronic endometritis, as it is called, pathological conditions external to the canal are still more easily recognized and are invariably found, so the same argument holds good as in reference to the acute form. But although the author believes endometritis as a distinct disease is rare, the endometrium has the power of very rapid and thorough absorption. Hence, it is through and by means of this membrane, undoubtedly, that septic material and irritating substances which reach the canal are frequently carried into the body of the uterus and to its adnexa and ligaments, where they cause an inflammation of more or less extent and distinctive character. The endometrium, from its anatomical connection with the uterus, must likewise become involved, but this involvement is a secondary one always in importance. The author queries: What is more probable than that this organ should endeavor to rid itself, by means of its glands, of the stasis in its venous circulation? And this is his belief, in that he considers uterine "leucorrhea" to be nothing more, in the majority of cases, than a symptom of some form of inflammation external to the endometrium, and usually external to the uterus as well, and as significant alone of an effort on the part of nature to find relief from a blood-stasis. This view of the subject received clinical confirmation from the experience of most physicians who have constantly cured completely all the symptoms ascribed to this supposed disease by applications of astringent drugs to the vaginal vault alone, without ever suturing the uterine canal. The author puts forth this remarkable statement: "That, with large opportunities for observation both in my own practice and that of others, I have never seen a case cured by curettage, unless in those diseases already excepted in the beginning of this article, viz.: retained placenta or secundines, acute sepsis from operative interference and the like, or in acute gonorrheal infection. The symptoms have always steadily returned, after a greater or less interval, when local treatment has ceased with the curetting." Finally, to support his position, the author offers the dicta upon this subject of one of the world's greatest pathologists, appending also the notes of Professor Welsh, of Johns Hopkins University, on endometritis.

---

### SOCIAL AND PROFESSIONAL VISITS.

The young man entering practice is often in a predicament and has hard work to make fine distinctions. One of his difficulties is in knowing how and when to make friends of his patients and patients of his friends. An older physician once said that for every friend made by a young man two patients were lost.

There is no doubt that the familiarity which springs from a close acquaintanceship between physician and friend soon begets a certain amount of contempt, and in too many cases the physician does better to hold himself aloof from too close communion with possible patients. The danger of making friends of patients is also emphasized when the visit which is on the dangerous borderland between social and professional is viewed from different sides by patient and physician.

A physician goes to see a sick one, be it man, woman or child, and he is asked to remain, perhaps to spend the evening or to take a meal. That is the fatal step. As soon as he begins to drop in in a familiar way to ask a few questions and make a few suggestions, and then remain for a social talk, just so soon is his position as family physician in jeopardy. Of course, there are exceptions to this statement, when the physician has reached the age of extreme maturity, and then comes the difficulty of turning him off when the family likes him as a friend, but feels that as a physician a better man might be found. When the time for rendering bills comes around then the position of both parties is usually clearly defined.

A young physician in New York State has just brought suit against a fair patient for a bill for services, which she maintains she does not owe, as so many of the visits were of a social nature. By bringing suit the physician made clear his position and found an enemy in his fair patient, and being in a small place, he probably hurt himself more than the amount of the bill, while the patient was taught the lesson that good services may be rendered, even though harmless talk and persiflage be mingled with the good advice. Still, such combinations are rather dangerous, and physicians especially are much more appreciated if they have little to say and refuse to converse on medical topics before a general audience or take up social chit-chat on an equality with the patient during a professional visit.

More misunderstandings arise from this lack of business methods than from many other causes. As long as there is illness in a house social visits should cease, and when professional advice is asked during a social visit it should either be given cheerfully and openly, so that the patient understands it is free, or else it should be made clear that it is a professional service.

It takes more than mere medical knowledge to make a successful physician, as the successful man with tact and a knowledge of human nature so well knows.—*Maryland Med. Journal.*

---

### "CHRISTIAN SCIENCE" AND SCIENCE.

It seems as if every age must have its fad, and perhaps we should not disquiet ourselves too much about it. Long ago the question was asked why the heathen raged and the people imagined a vain thing. The question, especially the latter part of it, is equally pertinent to-day; and the answer we venture to suggest is, because they like it.

The great beauty and merit of Christian science in the eyes of its devotees is that it affirms the thing that is not and denies the thing that is. It has to make grudging concessions to the law of gravitation and a few ordinary primary conditions of existence. In a kind of way it admits that certain injuries to the bodily frame may impair activity and even destroy life. That a man cannot walk without legs or do much useful thinking without his head are propositions which it has not yet seen its way to combat; but it takes its revenge on the system of visible things by comprehensive denials in a host of matters only a little less indisputable. It scornfully refuses to recognize pain or functional irregularities of any kind. Fevers, indigestions, inflammations, and the whole tribe of maladies which challenge the physician's art have no foundation in reality, and only need to be suitably ignored in order to be put to flight. If Job of old could only have planted himself at the Christian science point of view, he could have got rid of his boils in short order, and perhaps saved himself from the interminable and not overcheerful discourses of his friends. The great remedy, as recommended to-day in Christian science circles, is not to think about these things at all, and in case you cannot *think* hard enough, to send for a Christian science adept to help you. The adept will then, with cheerful and enthusiastic mendacity, inform you that you haven't any pain, that you haven't any boils, that you haven't any rheumatism or sciatica, or whatever it may be; and if you should point ruefully to the affected part, will exclaim: "Why, that isn't you; that's a mere mass of matter—and you are a soul, a spirit. You ought to rule your matter and not let your matter rule you." This is a point in the proceedings at which the faith of the sufferer is sometimes severely tried. Cases have been known in which, breaking into language neither wholly Christian nor rigorously scientific, the patient has demanded to know why, if that wasn't him—even grammar may be sacrificed in these emergencies—he should be enduring such abominable tortures on account of it; and up to date the satisfactory answer of Christian science to that particular question has not been formulated.—*Popular Science Monthly.*

---

### THE ANTI-VIVISECTION CRAZE.

It is gratifying to note an occasional protest against the passage of the bill to prevent vivisection in the District of Columbia in the secular papers. A few months ago the newspapers, with few exceptions, appeared to favor the bill, but as its real objects become better understood the sentiment against it appears to be growing. It was with a feeling akin to surprise that we read the following able protest against the bill in the *Times-Star* a few evenings ago:

"The same spirit that forced Socrates to drink the deadly hemlock, that compelled Galileo to retract his assertion that the world moved, that made Christopher Columbus a butt of ridicule for many years before he discovered America, that threw every possible obstacle in the way of the great scientist Harvey, and that made it criminal for physiologists hungering for knowledge to dissect a corpse, is now at work in the United States Congress seeking to prevent any form of vivisection. The spirit is prompted by ignorance. It comes from a lot of misguided fanatics of the same kidney as those who insist that vaccination against smallpox is vain, that all bacteriologists are humbugs. It is the plan of these fanatics to pass a bill through the United States Congress forbidding vivisection in the District of Columbia, and use this as an entering-wedge for further legislation in the various States of the Union. As yet they have failed to prove a single case of cruelty in the conduct of animal experimentation in the District. Still they seek to establish a system of espionage upon all physicians and scientists who seek to enlarge the knowledge of the world upon many things pertaining to life and health that are still hidden and vital. The commission which these fanatics urge is to be made up of non-professional persons, who are to be given the power to judge as to motives and methods of scientists. It is the purpose to make masters out of tyros, to let the blind lead those who can see.

"The same spirit of fanaticism forced Lord Lister, who has probably done more to alleviate the sufferings of the wounded than any other thousand scientists of the present century by his discovery of antiseptics, to leave England and go to the continent of Europe to push his experiments. As a result of the Lister discoveries, blood-poisoning in cases of surgery is now unnecessary. During the last war in this country blood-poisoning was responsible for at least seventy-five per cent. of the deaths of those who were subjected to surgery. Experiments in vivisection made Lister's triumph possible. One of these anti-vivisectionists recently stated that his design was to stop even the hypodermic puncture of an animal. Carrying the purpose of these people to their legitimate conclusion, all physiological work would be stopped. No bacteriological experiments could be made. No tests as to relative value of suture materials, no new abdominal operations could be devised and tried beforehand, no more diphtheria serum could be manufactured in this country, nor could we obtain any more vaccine virus from animals; neither could we consistently import any of the animal serums or virus for use here, as the importation of these articles would manifestly create a necessity for the use of the animals abroad to secure the material.

"There is no scientific man in this country of any prominence who favors the bill. The purposes of vivisection experiments, carried on as they are in the majority of cases, are not to gratify curiosity, but to restore the health and prolong the life of human beings. To oppose them, as the pending bill proposes, is to co-operate with disease and with death."—*Ohio Med. Jour.*

---

### A Decision of Importance to Physicians.

A case of interest to physicians generally was decided by Judge Dunbar, at Boston, last week. The circumstances of the case, as reported were that previous to May 1, 1896, Dr. Oscar F. George had a lucrative practice in Lynn. On that date he sold it to Dr. Edward B. Herrick, who came from Amherst, Mass., signing an agreement not to practice in the city as long as Dr. Herrick remained there. He then went to Newburyport and later to Vermont, and about March 1, 1897, came to Swampscott, where he located and resumed practice, and, as he admitted upon the witness-stand, again began practice among his old patients in Lynn. Dr. Herrick brought a bill in equity in the superior court against Dr. George, to have him restrained from practicing in Lynn. As a result Judge Dunbar enjoined the defendant from practicing in Lynn in violation of his promise. The decision is important from the fact that while the defendant admitted that morally he was bound to keep his agreement, legally he was not so bound. The judge, however, decided that he was both legally and morally bound to keep his agreement, and enjoined the defendant from further trespassing upon the ground to which he had signed away all claim.—*Boston Med. and Surg. Journal.*

### MALARIA AND THE CUBAN CAMPAIGN.

Summing up an interesting editorial on the above subject in the *Monthly Cyclopedia of Practical Medicine*, Dr. Charles E. Sajous concludes:

The following prophylactic measures, carried out simultaneously, become necessary in malarial districts to insure adequate protection:

1. *To avoid contamination through the respired air and inoculation by insects.*—Unacclimatized men, white or black, should not be employed for the digging of trenches, the erection of defenses, or any other kind of work involving upturning of the soil. Natives should alone be utilized for this work.

High ground should be selected for camp-sites, windward, if possible, of any swamp, pool, stream, etc., that may be in the neighborhood.

The men should sleep as high above ground as possible (not less than two feet, and if practicable, from twelve to fifteen feet) and be provided with mosquito netting.

While crossing malaria-laden forests, glens, lowlands, swamps, etc., the men should be ordered to avoid talking.

2. *To avoid contamination by water.*—When water from malarial regions is alone available for drinking purposes, it should be filtered or, preferably, sterilized by boiling.

Bathing should not be permitted when water from a malarial region can alone be obtained, but washing of the body with such water is permissible, provided carbolic-acid soap be employed.

3. *To prevent the development of malarial parasites in the blood.*—Four grains of hydrochlorate of quinine should be administered morning and evening during meals as prophylactic, beginning two days before the malarious region is reached.

4. *To conserve the general powers of resistance of the economy.*—Regular and frequent periods of rest should intersperse long marches. Drenching and wading through streams should be avoided when possible. Varied and adequate food should be furnished.

The head should be so protected as to secure a maximum amount of coolness under all degrees of temperature, a head-gear such as the solar tepé being furnished for this purpose.

5

### RESULTS IN 100 ABDOMINAL OPERATIONS.

Shoemaker (*Phila. Med. Journal,* March 26) gives the results of an investigation in a consecutive group of celiotomy cases of various sorts, all the cases being women with gynecologic disorders. Of the 100 cases 6 died; 2 cases of hysterectomy for fibromata weighing fifteen and one-half and seven and one-half pounds respectively; 1 case of extra-uterine fetation, septic when first seen; 1 following removal of ovaries in intraligamentary fibroid tumor of uterus, adhesive to pelvis; 1 following removal in severe double gonorrheal pyosalpinx with bowel adhesions, and one, unexpected, following removal of one diseased ovary and tube and suspension of retroverted uterus. Hysterectomy was done 19 times. In the series 3 were malignant, 8 fibromas, 1 a fibrocyst in an extra-uterine case, 7 were for hopeless inflammatory disease of uterus, tubes and ovaries. He believes that "the uterus is better removed when itself diseased, if large, heavy and retroverted, with poor support, when it has been for years the channel for outpour for chronic discharges, when hemorrhage has been excessive from glandular degeneration of the endometrium. This is especially true in elderly multiparæ. The risks of removal are not great." He, however, is opposed to removal of normal ovaries for nervous conditions, believing in correction of the disease causing the persistent irritation or hemorrhage, as a means of relieving the nervous condition. Thirty-nine cases with gross anatomic lesions showed marked nervous disturbance, and 77 per cent. are cured or markedly improved. In numbers of cases marked hysteric or other nervous disturbance proved to be only a surface-play of symptoms, while serious pelvic disease had been one of a chain of causes. Hernia in operation wounds occurred six times, in one case a woman, operated on when four months pregnant, who went safely through delivery at term, the hernia occurring nineteen months later. Inflammatory disease of tubes and ovaries, requiring removal, occurred thirty-five times, extra-uterine pregnancy was present five times, cystic tumors of the ovaries seven times; in one case there was a large dermoid; broad-ligament cysts were present three times; five cases had abdominal tuberculosis. Uterine suspension was done but once as an independent operation for severe symptoms due to retroversion and descent of the uterus. This patient was cured permanently and has remained well more than two years after ten years of suffering. Of the ninety-four cases surviving operation, 49 (59 per cent. of the cases heard from) are cured after a period of from six months to two years; thirty-two are improved, though fourteen of these are anatomically cured; four of the five tuberculous cases were improved and one cured. The most satisfactory results were in the large tumor cases, then the chronic pus cases in which there was one death in twenty-two cases. He concludes: "As to benign tumors, there are none involving uterus, tubes and ovaries that cannot, at the present day be removed with a comparatively low mortality, depending upon the stage at which the case is seen and upon the skill and experience of the operator in abdominal work, on his surgical judgment and on the organization of assistants and plant, with the aid of which he operates. Very much depends upon good detail work. By the modern steam sterilizer the last excuse for a septic death has been removed. Even the stitch-hole abscess should be rarest of exceptions and call for rigid explanation."

---

### THE SCIENTIFIC AMERICAN NAVY SUPPLEMENT.

The *Scientific American*, which has always identified itself very closely with the interests of the navy, is to be congratulated on the extremely handsome and valuable "Navy Supplement" which it has lately put before the public. We think that if the average reader had been asked beforehand what kind of a work he would prefer upon the Navy, he would have asked for just such an issue as this.

Both the illustrations and the reading matter are of the straightforward explanatory kind which is necessary to put a technical subject clearly before the lay mind. It was a happy thought to preface the work with a chapter upon the classification of warships and insert a few diagrams by way of explanation of the subtle differences between cruisers, monitors and battleships, for after digesting this chapter one is prepared to follow intelligently the detailed descriptions of the various ships which make up the bulk of the issue. One of the best things about this number is that it does not merely give an external illustration of each ship, but it takes the reader down below decks, and initiates him into the mysteries of the magazines, handling rooms, ammunition hoists and motive machinery. The sectional views of the interior of the turrets of the monitors are exceptionally fine, as is the large wood engraving of the engines of the "Massachusetts." The last page of the number contains complete tables of the new navy, the auxiliary fleet and the various naval guns. A handsome colored map of Cuba and the West Indies is furnished with this issue. We extend our congratulations to our contemporary on the production of a work which is well conceived and admirably carried out. This work is published by Munn & Co., of 361 Broadway, New York, for 25 cents.

---

### FRACTURE OF THE NECK OF THE FEMUR IN CHILDHOOD.

Whitman (*Annals of Surgery*) reports ten cases of fracture of the neck of the femur in children between two and a half and eight years of age, and presents two skiagraphs showing the consequent deformity of the bone at the seat of the injury. In the course of this paper, the author deals with certain practical difficulties in connection with this injury, especially its immediate diagnosis, and its differential diagnosis from hip disease during the stage of recovery. It is held that the traditional age limit for fracture of the neck of the femur does not exist, and that this injury not only does occur in childhood, but is probably not an uncommon accident which may be recognized, or may at a later period be mistaken and treated for hip disease. In opposition to the accepted teaching that the symptoms here regarded as those of fracture are due to separation of the epiphysis of the head of the femur, the author holds that it would be rather extreme violence followed by non-union of the fragments or subsequent disability that would favor the diagnosis of the latter form of injury. The less the violence and the less the immediate disability, the greater would be the probability of fracture when signs of fracture are present, because a bone is more likely to break in its weakest than in its strongest part, and because rapid union and almost normal functional capacity associated, as was observed in all the author's cases, with upward displacement of the trochanter, prove that the solution of continuity could not have involved the articulating surface of the femur.—*British Medical Journal.*

---

### BRAIN ABSCESS IN INFANTS.

In the *Archives of Pediatrics* for February and March, Holt reports five cases of abscess of the brain in infants and a summary of twenty-seven collected cases in infants and very young children. He concludes that: 1. The affection is rare under five years of age. 2. Otitis and traumatism are the principal causes. 3. It most often follows neglected cases, though rare in acute otitis, and is usually secondary to disease of the petrous bone. 4. In cases in infancy without evident cause, the ears are probably the source of infection. 5. Abscess rarely develops after injury to the head without fracture of the skull, and cerebral symptoms arise within two weeks after the injury in nearly all traumatic cases. 6. Only general symptoms are present in a large proportion of the cases. 7. Unless they are constant, focal symptoms may be misleading, and even when constant may depend on meningitis or other associated lesions. The motor symptoms alone can be relied on. 8. Rapid progress, fever, and history of injury generally diagnostic from tumor, while lumbar puncture assists in slower cases with little or no fever. 9. Where there are only terminal symptoms, the diagnosis from meningitis is impossible. In the more protracted cases the distinctive points are the slower and more irregular course and generally a lower temperature. 10. Operation should not be urged unless definite localizing symptoms, the principal one hemiplegia, are present.—*Jour. Am. Med. Assocn.*

---

### ANEMIA.

Jennie M———, Stamford; aged 30; first seen January 29, 1898. Anemia and general malnutrition; blood profoundly lowered in quality, and all the usual symptoms of a well-defined case, such as pale gums, waxy complexion, loss of flesh, and general functional disturbance. Patient suffered nightly with agonizing neuralgia.

Her digestion was so upset, that I put her on only twenty drops bovinine hourly, in iced lime water to correct acidity. This was well retained, but did not satisfy hunger. On the 21st gave a rectal injection of eight ounces: three of bovinine and five of milk. This was retained, and the night passed without the neuralgia, so that she awoke greatly refreshed and improved. A drachm of bovinine in milk and lime water was now given every two hours, and was retained, with a slight gaseous disturbance, and an enema of two ounces of bovinine and four of milk at night, which was retained, and this night also passed without the neuralgia. This treatment was continued to February 16th, when improvement was so far advanced that the rectal injection was discontinued, and a tablespoonful of bovinine in old port wine, alternated with milk, was comfortably taken per mouth every three hours, until the 10th; when the bovinine was again increased to a wineglassful in milk every three hours, and so taken until the 19th. At this time, microscopical examination of the blood showed very little shortage of red cells and hemaglobin, and externally the patient presented every appearance of restored health. On the 20th, the cure was pronounced complete and case discharged.

---

### Diabetic Albuminuria and its Treatment.

Goudart has recently devoted much attention to this subject (*British Medical Journal.*)

First, the frequency of albuminaria in diabetes is variable, and may occur in two forms, functional and that due to grave nephritic disease. In the first form it may be extremely slight, or else constitute a very marked feature in the case. When slight, proper dieting and small does of antipyrine combined with a little bicarbonate of soda in the form of a powder may be given every one and a half hours before each meal. This treatment should not be continued more than three or four days, beyond which time the antipyrine will become injurious. It is well to prescribe some quinine, wine and Vichy water at meals. After this treatment the sugar decreases considerably, in other cases it remains unaffected.

In the first instance, anti-diabetic treatment may be set aside and attention devoted to the albuminuria; in the second instance, it is advisable to order small doses of arseniate of soda combined with codeine and carbonate of lithia. Most usually the glucosuria diminishes under this treatment, and the albuminuria is then treated in the same manner as above. This line of treatment is usually followed by extremely satisfactory results. After a fortnight or so it is recommended to give phosphates with nux vomica, or later, hypophosphites of lime, potash, or soda with quinine. Should the quantity of albumen eliminated in twenty-four hours reach two or three grammes the case is practically one of Bright's disease, and patient is put on milk diet. The author now recommends lactate of strontium in small doses.—*Charlotte Medical Journal.*

---

### WOODBRIDGE TREATMENT OF TYPHOID.

Rudolph W. Holmes (*Philadelphia Medical Journal,* January 15, 1898) reports twenty-six cases of typhoid fever treated by the Woodbridge method under Woodbridge's direct supervision, and arrives at the following conclusions: (1) Woodbridge treatment does not abort. (2) The mortality is not influenced by the treatment. (3) Five to eight per cent. of typhoids in an epidemic of mild type or medium severity will cure themselves within two weeks. (4) A user of the Woodbridge treatment who invariably has abortive results does not correctly diagnose *all* his cases. (5) By the Woodbridge treatment complications are not affected. (6) An immediate, positive diagnostic aid is prerequisite to making statements concerning any abortive treatment valuable. (7) Believers in the abortive treatment of typhoid must bear in mind the existence of the *abortive form of Liebermeister* and the *typhus levis* of Griesinger to intelligently differentiate typhoid from the diseases with which it may be confounded.—*University Med. Magazine.*

---

### CARE OF THE SICK AND WOUNDED.

It has been decided that the naval ambulance ship Solace shall be used as a transport for the sick and wounded of both army and navy. She will carry men physicians disqualified for active service from the fleet or from Cuba to Key West and Tampa. A hospital train will run from Tampa to northern points in order to give the sick a benefit of a change to the cooler climate of the middle and northern Atlantic seaboard. A general army and navy hospital will be established at Key West, and hospital tents will be sent there to accommodate any overflow of incapacitated seamen and soldiers. The selection of Key West for this general hospital is due chiefly to the fact that the island is more healthful than places on the mainland. In the event of a fever outbreak among the men in Cuba or on the warships, the treatment of the stricken there would lessen the danger of a spread of the disease to the coast proper. Other hospitals are to be established in the department of the Gulf, but that at Key West will be the headquarters of the medical corps of both military services, in addition to the marine hospital service.—*Med. Record.*

---

### TO DETERMINE THE SEX OF THE CHILD IN UTERO.

The following method is about as reliable as any:

Dr. X., of Paris, has discovered an infallible method of determining the sex of the child *in utero.* After a conscientious auscultation and palpation of Madame Z., he announces that it will be a boy, and at the same time notes on his tablets: "Madame Z.—a girl." When the accouchement takes place, if the newcomer is a boy, well and good; if it is a girl, he exhibits his tablets and assures the mother that she must have misunderstood him.—*Ex.*

---

Do not cauterize infected wounds unless it is to obtain a moral effect in a scared patient. It was shown more than fifty years ago that when horses were inoculated with glanders, and sheep with pox, cauterization with red hot iron, applied ten minutes after inoculation, failed to check the disease. An infected wound should simply be well laid open and covered with a wet dressing. The use of nitrate of silver to cauterize wounds is a harmful absurdity.—*International Journal of Surgery.*

